# The Protective Effect of Bosentan against Atherosclerosis in Apolipoprotein E-Deficient Mice Is Mediated by miRNA-21

**DOI:** 10.1155/2019/8348430

**Published:** 2019-12-05

**Authors:** Xiaona Xu, Zhiqiang Zhao, Guangping Li

**Affiliations:** Tianjin Key Laboratory of Ionic-Molecular Function of Caidiovascular Disease, Department of Cardiology, Tianjin Instiute of Cardiology, The Second Hospital of Tianjin Medical University, Tianjin 300211, China

## Abstract

Vascular calcification is an independent risk factor for plaque instability and is associated with endothelial cell function. Here, we investigated the role of endothelial cell function in the calcification of atherosclerotic plaques. We hypothesized that atherosclerosis would be associated with endothelial dysfunction and that bosentan (Tracleer®), a dual endothelin-receptor antagonist, would preserve endothelial cell function in an apolipoprotein E-deficient (ApoE^−/−^) mouse model of atherosclerosis. Accordingly, 4–6-week-old ApoE^−/−^ mice were fed a high-fat diet and treated with bosentan, and the effects of this treatment on body weight and blood lipid concentrations was evaluated. Endothelial damage in the aortic arch was assessed immunohistochemically to detect the proapoptotic proteins PDCD4, caspase-3, and Bax and the antiapoptotic protein Bcl-2. Notably, bosentan treatment was associated with decreased concentrations of these proteins and of blood lipids in ApoE^−/−^ mice. Consistent with these findings, we observed increased concentrations of miRNA-21 and PDCD4 mRNA expression in the aortic arch endothelium after bosentan treatment. We conclude that bosentan can prevent endothelial cell death and protect against atherosclerosis in ApoE-deficient mice by upregulating miRNA-21.

## 1. Introduction

The pathobiology of atherosclerosis is characterized by the failure or malfunction of endothelial cells [1, 2], particularly in lesion-prone areas of the endothelial lining of the arterial vasculature. This lining interacts with the circulating blood and acts as an important transducer of mechanical and humoral signals [3–5]. In response to these signals [2, 6], vascular endothelial cells maintain vascular homeostasis via paracrine and autocrine mechanisms [7]. These interactions create a pathological risk of atherosclerosis, as phenotypic changes in the endothelium can lead to dysfunction. Moreover, the development of new drugs and treatments for atherosclerosis requires a more in-depth understanding of the endothelial biology of atherosclerosis [8].

MicroRNAs (miRNAs) comprise a broad class of small noncoding RNAs that modulate the expression of complementary target genes [9–13]. Dysfunction of miRNAs is associated with multiple pathological processes, including atherosclerotic vascular disease [14, 15]. Recent studies have confirmed that miRNA-21 is expressed in endothelial cells, macrophages, and smooth muscle cells [1, 16–18] and have identified a crucial role of this miRNA in the progression of diseases such as cancer and cardiovascular diseases [2, 16]. Furthermore, the importance of endothelial cells to atherosclerosis suggests that the expression of miRNA-21 in these cells may also contribute to the progression of disease. However, few previous studies have analyzed miRNA-21 expression patterns in atherosclerosis-related tissues.

Programmed cell death 4 (PDCD4) was recently identified as an important functional target of miRNA-21 [19, 20]. According to previous work, this protein acts as a tumor suppressor [21] and is upregulated during apoptosis. However, it remains unclear how miRNA-21 [22] regulates the specific and potentially pathological activities of PDCD4, such as proliferation and apoptosis. Therefore, clarification of the role of miRNA-21 (and other miRNAs) and its target PDCD4 in atherosclerosis is urgently needed. In this study, we generated an animal model of atherosclerosis by raising apolipoprotein E-deficient (ApoE^−/−^) mice on a high-fat diet and investigated the expression of miRNA-21 and its target PDCD4 during the progression of atherosclerosis in these animals.

## 2. Materials and Methods

### 2.1. Animals

All animal experiments were approved by and conducted according to the guidelines of the Animal Care and Use Committee of the Second Hospital. Wild-type C57BL/6J mice were purchased from the Jackson Laboratory (Beijing, China). All animals were housed in plastic cages (four mice/cage) under a 12 h light/dark cycle with free access to water and food. Bosentan was purchased from Sigma-Aldrich (St. Louis, MO, USA). Mice were randomly divided into two experimental groups, ApoE^−/−^ and ApoE^−/−^ + bosentan, and one control group (*n* = 10/group). All mice were treated with a one-week period of adaptive feeding before the start of the experiment. Subsequently, the control group was fed a basal diet (Fukang Biotechnology Co., Ltd. Beijing, China), while the two experimental groups were fed a high-fat diet containing 10% lard, 8% egg yolk powder, 2% cholesterol, 0.2% bile salt, and 80% basal diet (Fukang Biotechnology Co., Ltd.). Mice in the ApoE^−/−^ + bosentan group were administered with daily intragastric doses of bosentan (100 mg/kg body weight). Mice in the control and ApoE^−/−^ groups were administered with daily doses of normal saline via intragastric administration. All mice were sacrificed by cervical dislocation at week 12 of the experiment.

### 2.2. Collection of Samples and Determination of Hematological Parameters

Mice from different groups were anesthetized with ether and sacrificed by cervical dislocation. Blood samples were collected and stored at −80°C for subsequent biochemical studies. The blood concentrations of triglycerides (TG), high-density lipoprotein cholesterol (HDL), low-density lipoprotein cholesterol (LDL), and total cholesterol (TC) were measured using an Auto Hematology analyzer (Olympus Corporation, Tokyo, Japan).

### 2.3. Histological and Immunohistochemical Staining

To prepare specimens for microscopic imaging, aortic sinus tissues were dehydrated in a series of increasing concentrations of alcohol and xylene and subsequently embedded in paraffin. Next, 8 *μ*m-thick sections were cut from the central segments of the fixed tissues using a rotating microtome (Leica® RM 2145, Wetzlar, Germany) and mounted on SuperFrost Plus slides (ThermoFisher Scientific, Waltham, MA, USA). The aortic sinus sections were subjected to dewaxing and rehydration via consecutive immersion in xylene and graded ethanol solutions. Hematoxylin and eosin staining of the sections was performed according to standard methods. Immunohistochemical staining was performed as described by Kato et al. [23].

### 2.4. RNA Isolation and Quantitation

Total RNA was extracted from the aortic tissues and purified using TRIzol reagent (Invitrogen, Carlsbad, CA, USA). One microgram of total RNA per sample was then used in a quantitative polymerase chain reaction (qPCR) analysis. Reverse transcription was performed using the M-MLV Reverse Transcription system (Takara Co. Ltd., Dalian, China) under the following conditions: 42°C for 2 min, 95°C for 5 s, and 37°C for 15 min. The resulting cDNA was subjected to qPCR using the SYBR Green reagent (Takara Co. Ltd.) and an ABI 7500 quantitative PCR instrument (Applied Biosystems, Inc., Foster City, CA, USA) under the following conditions: pre-denaturation at 95°C for 10 min, followed by 35 cycles of denaturation at 95°C for 15 s, annealing at 60°C for 25 s, and extension at 72°C for 30 s. Three independent reactions were run per sample. The relative mRNA concentrations were calculated after normalization to the concentration of GAPDH mRNA expression (internal control). The following primers were used: mmu-miR-21-Fwd: 5′-GTCAGGC TAGCTTATCAGA-3′; U6-Fwd: 5′-CTCGCTTCGGCAGCACA-3′, U6-Reverse: 5′-GTATCCAGTGCAGGGTCCGAGGT-3′; PDCD4-Fwd: 5′-AGGTCGTCTTAAACCAGAGAG-3′, PDCD4-Reverse: 5′-ATGTCAGAAATGCCTTGTACC-3′, GAPDH-Fwd: 5′- TCAAGAAGGTGGTGAAGCA-3′, GAPDH-Reverse: 5′-GTCAAAGGTGGAGGAGTGG-3′.

### 2.5. Statistical Analysis

All statistical analyses were performed using Prism 8 software (GraphPad, Inc., La Jolla, CA, USA). All results are expressed as mean values ± standard deviations. A *P* value of <0.05 was considered to indicate statistical significance, and clear statistical differences are commonly indicated by asterisks in figures (e.g., ^*∗*^*P* < 0.05). Student's *t*-test was used for comparisons of two groups. A one-way ANOVA and the Bonferroni posttest were used for comparisons of more than two groups.

## 3. Results

### 3.1. Histopathological Analysis of Atherosclerosis in ApoE^−/−^ Mice Fed a High-Fat Diet

**Figure 1:**
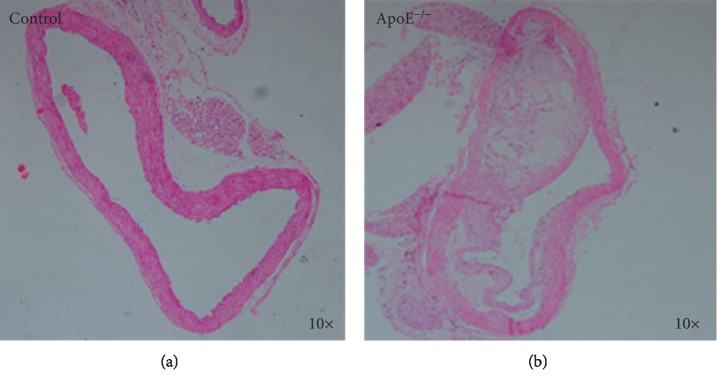
Key histological features of tissue from an ApoE^−/−^ mouse. Histological sections of aortic sinus tissues from control and ApoE^−/−^ mice were stained with hematoxylin-eosin. ApoE^−/−^ mice developed typical atherosclerosis of the aortic arteries in response to a high-fat diet.

At week 12, mice from the control group and ApoE^−/−^ groups were sacrificed, and the aortic sinus tissues were collected. The successful inclusion of atherosclerosis was shown in the ApoE^−/−^ group. The ApoE^−/−^ mice presented with features of typical atherosclerosis, such as a thin fibrous cap and the presence of foam cells and cholesterol crystals within the atherosclerotic plaque (Figure 1).

### 3.2. Bosentan Treatment Did Not Affect Blood Lipid Concentrations in ApoE^−/−^ Mice

Mice in the ApoE^−/−^ and bosentan-treated groups exhibited significantly larger gains in body weight relative to mice in the control group (Figure 1). Moreover, ApoE^−/−^ mice exhibited a significantly greater weight gain than those in the bosentan-treated group (Table 1).

As shown in Table 2, a hematological analysis revealed lower concentrations of TG, HDL, LDL, and TC in the control group relative to the ApoE^−/−^ group. These indices differed significantly between the control group and bosentan group but not between the ApoE^−/−^ and bosentan groups.

### 3.3. Histopathological Analysis of Atherosclerosis in ApoE^−/−^ Mice Treated with Bosentan

**Figure 2:**
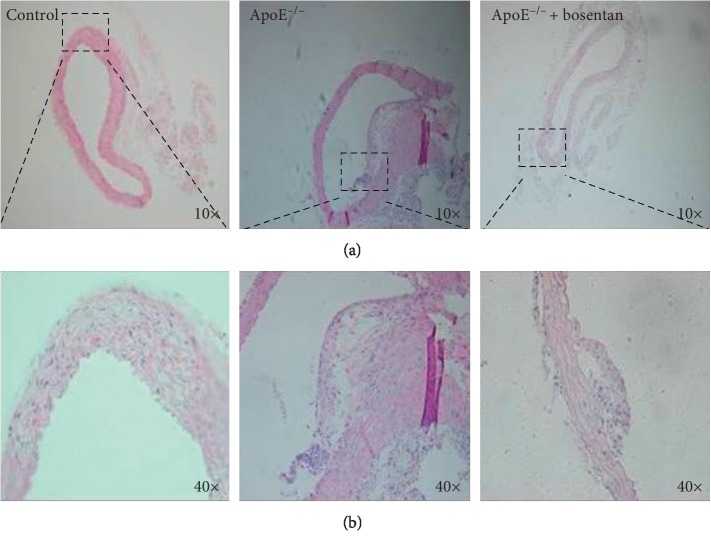
Treatment with bosentan reduced atherosclerotic plaque formation in ApoE^−/−^ mice. (a) Hematoxylin-eosin staining of atherosclerotic plaques in bosentan-treated ApoE^−/−^ mice. T atherosclerotic plaques contained fewer foam cells and cholesterol crystals than those from untreated mice (magnification: 10X). (b) Enlarged sectional areas of plaques in the control, ApoE^−/−^, and bosentan groups (magnification: 40X).

At week 12, mice from the control, ApoE^−/−^, and bosentan groups were sacrificed, and the aortic sinuses were collected. As noted above, HE staining revealed characteristic features of atherosclerosis in the aortic sinuses of ApoE^−/−^ mice. Notably, although typical atherosclerosis was observed in the bosentan group, these mice had significantly smaller atherosclerotic plaques than those observed in the ApoE^−/−^ mice (Figure 2). The observations suggested that bosentan played a protective role against atherosclerosis in ApoE^−/−^ mice raised on a high-fat diet.

### 3.4. Bosentan Treatment Reduced the Population of PDCD4-Positive Cells in ApoE^−/−^ Mice

**Figure 3:**
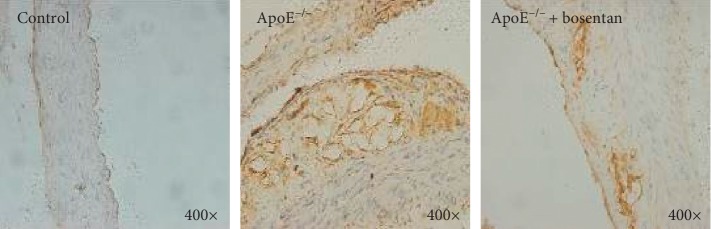
Bosentan treatment reduced PDCD4 expression in aortic sinus tissues from ApoE^−/−^ mice. Immunohistochemistry staining for PDCD4 in atherosclerotic plaques from the indicated groups of mice.

We further investigated the expression of PDCD4, a proapoptotic protein, in aortic sinus sections from the three groups of mice. Immunohistochemistry staining of the aortic sinus sections for PDCD4 revealed a significantly larger population of PDCD4-positive cells in ApoE^−/−^ mice compared to the control group (Figure 3). Interestingly, although more PDCD4-positive cells were observed in the bosentan-treated group relative to the control group, this cell population was significantly smaller in the bosentan-treated group relative to the ApoE^−/−^ group (Figure 3). Accordingly, it appeared that bosentan might have reversed the endothelial cell death induced by a high-fat diet in ApoE^−/−^ mice.

### 3.5. Bosentan Treatment Reduced Caspase-3 and Bax Expression and Increased Bcl-2 Expression in the Aortic Sinuses of ApoE^−/−^ Mice

**Figure 4:**
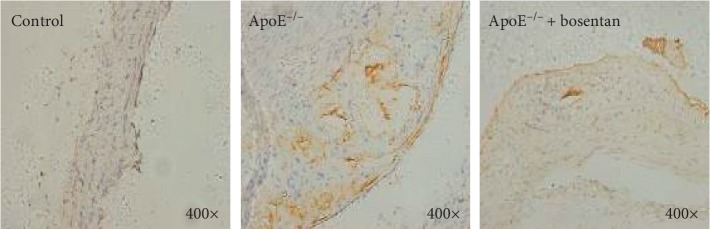
Bosentan reduced caspase-3 expression in the aortic sinuses of ApoE^−/−^ mice. Immunohistochemistry staining for caspase-3 in atherosclerotic plaques from the indicated groups of mice.

**Figure 5:**
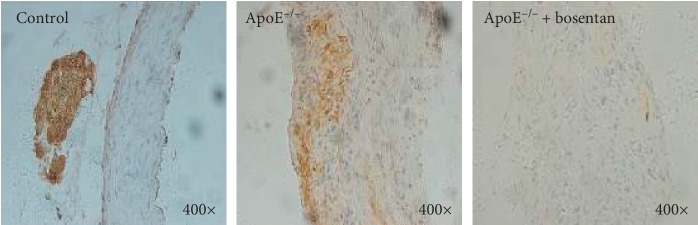
Bosentan reduced Bax expression in the aortic sinuses of ApoE^−/−^ mice. Immunohistochemistry staining for Bax in atherosclerotic plaques from the indicated groups of mice.

**Figure 6:**
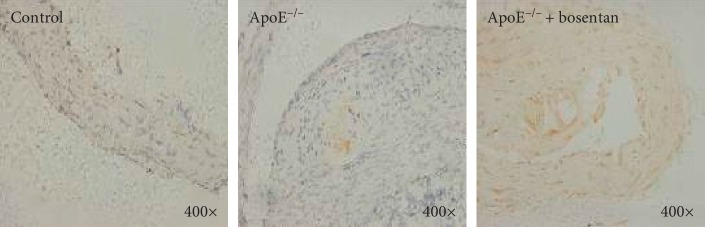
Bosentan increased Bcl-2 expression in the aortic sinuses of ApoE^−/−^ mice. Immunohistochemistry staining for Bcl-2 in atherosclerotic plaques from the indicated groups of mice.

To further confirm the antiapoptotic role of bosentan in the vascular endothelial cells of ApoE^−/−^ mice fed a high-fat diet, we performed immunohistochemistry staining to detect caspase-3 (a downstream effector of Bcl proteins in the apoptotic cascade), Bax (a proapoptotic Bcl family member), and Bcl-2 (an antiapoptotic Bcl family member) in the aortic sinus tissues. As expected, we observed increases in caspase-3 and Bax positivity in the aortic sinuses from the ApoE^−/−^ group relative to the control group. In contrast, bosentan treatment significantly reduced the concentrations of caspase-3 and Bax in the atherosclerotic plaques of ApoE^−/−^ mice (Figures 4 and 5). More importantly, mice in the ApoE^−/−^ group exhibited higher Bcl-2 concentrations in the aortic sinus when compared with control mice, whereas bosentan treatment significantly increased the expression of Bcl-2 in the atherosclerotic plaques (Figure 6).

### 3.6. Bosentan Treatment Enhanced miRNA-21 Expression and Decreased PDCD4 mRNA Expression in ApoE^−/−^ Mice

**Figure 7:**
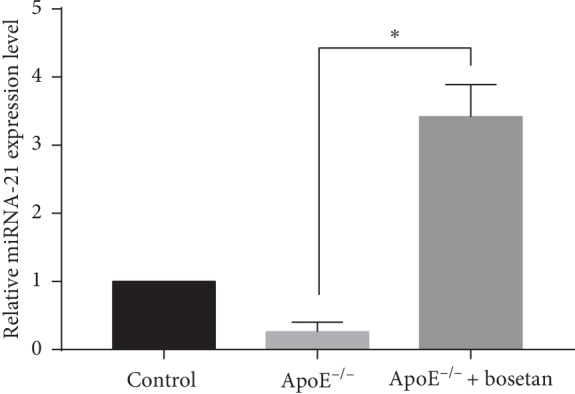
Bosentan enhanced the expression of miRNA-21 in ApoE^−/−^ mice. A real-time qPCR analysis of miRNA-21 expression in atherosclerotic plaque from the indicated groups of mice. The miRNA abundance in each group was normalized to U6 RNA (internal control).

**Figure 8:**
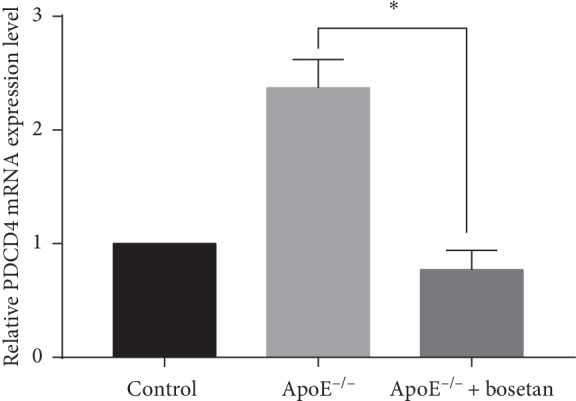
Bosentan reduced the expression of PDCD4 mRNA in ApoE^−/−^ mice. A real-time qPCR analysis of PDCD4 mRNA expression in atherosclerotic plaques from the indicated groups of mice.

MiRNA-21 targets the gene encoding PDCD4. Therefore, we next investigated the expression of miRNA-21 and PDCD4 mRNA in the aortic sinus. As shown in Figure 7, we observed a decreased concentration of miRNA-21 expression in the ApoE^−/−^ group relative to the control group, whereas bosentan treatment significantly increased the expression and resultant concentration of miRNA-21. Consistent with the immunohistochemistry data, we also observed a significant increase in the expression of PDCD4 mRNA in the ApoE^−/−^ group relative to the control group, whereas bosentan treatment induced a dramatic decrease in PDCD4 expression (Figure 8). Our data suggest that the antiapoptotic effect of bosentan on endothelial cells may be mediated by miRNA-21.

## 4. Discussion

The Tampere Vascular Study [24] recently completed a series of miRNA expression profiles in human tissues. This study of femoral atherosclerotic, aortic, and carotid arteries acquired 12 atherosclerotic plaques and determined that miRNA-21 was the most strongly detected miRNA in the femoral artery samples. Notably, significant increases in miRNAs-21, -34a, and -210 were observed in the carotid artery samples relative to the control. The atherosclerosis-related functions of miRNA-21 are known to be associated with the inhibition of matrix metalloproteinases (MMPs) and the proliferation of vascular smooth muscle cells (VSMCs) [25]. Previous reports described the prominent role of miRNA-21 in VSMC proliferation [26, 27]. Moreover, the genetic and pharmacological inhibition of miRNA-21 expression was shown to significantly reduce the incidence of balloon-induced neointimal carotid artery injuries [20]. These findings have stimulated research exploring the manipulation of miRNA-21 expression as a possible local therapeutic strategy. However, the molecular mechanism by which bosentan regulates miRNA-21 remains unclear and requires further investigation. In this study, we demonstrated that bosentan may be an effective treatment in patients with atherosclerosis, particularly as this drug led to major reductions in the atherosclerosis plaques of ApoE-deficient mice *in vivo*. This effect was likely attributable to the miRNA-21-mediated upregulation of antiapoptotic Bcl-2 and the downregulation of PDCD4 and the downstream proapoptotic effectors caspase-3 and Bax, resulting in a net antiapoptotic outcome.

Atherosclerosis, a major risk factor for coronary heart disease, is caused by the accumulation of cholesterol, macrophages, and cellular waste products (e.g., calcium and fatty materials) on artery walls. These deposits increase the thickness of the affected artery wall and can radically restrict blood flow [28, 29]. These physiological changes are associated with the pathology of atherosclerosis [30], wherein lipoproteins transport lipids in the blood and deposit these substances on arterial walls. Notably, we observed significant increases in the concentrations of TC, TG, and non-HDL-C and in the sizes of atherosclerotic lesion areas in ApoE^−/−^ mice fed a high-fat diet, compared to mice fed a basal chow diet [28]. These phenomena indicated that dysregulation occurs in the sphingolipid and glycerophospholipid metabolic pathways during atherosclerotic dyslipidemia [31]. Moreover, we observed significant increases in the serum TG, TC, LDL, and HDL concentrations in both the bosentan and ApoE^−/−^ groups relative to the control group. These data strongly suggested that certain dietary factors, such as high-fat diets, can enhance the development of atherosclerosis.

PDCD4 has been associated with biological processes such as inflammation and apoptosis [32, 33]. Notably, endothelial damage and dysfunction are the primary factors underlying atherosclerotic changes. In a previous study, PDCD4 deficiency greatly enhanced the ability of oxidized LDL to impair the autophagy efflux, which prevented the differentiation of macrophages to foam cells [34]. Another study determined that the downregulation of PDCD4 expression in the diseased vascular wall could cause an imbalance between VSMC apoptosis and proliferation, suggesting that PDCD4 may be a therapeutic target for proliferative vascular disease [35]. In atherosclerosis, PDCD4, as a target of miR-16, may inhibit the activation of inflammatory macrophages via the mitogen-activated protein kinase (MAPK) and NF-κB and signaling pathways, suggesting that PDCD4 could be a focus of atherosclerosis therapy [36].

The apoptosis-related protease caspase-4 has been identified in atherosclerotic plaques [37], whereas active caspase-3 was not detected in the neointima of normolipidemic animals after arterial injury [38]. In another study of caspase-3 expression, overexpression of the miRNA has-let-7 g was identified as key to the negative regulation of apoptosis in EAhy926 endothelial cells [39]. In apoptotic cells, morphological features such as DNA cleavage, nuclear condensation, and plasma membrane blebbing are blocked directly by Bcl-2, a negative regulator of cell death [40]. Bcl-2 is involved in cell survival and inhibits cell death in response to chemotherapeutic agents and stimuli such as ethanol and heat shock. In our study, we observed fewer PDCD4−, caspase-3−, and Bax-positive cells and more Bcl-2-positive cells in bosentan-treated mice relative to ApoE^−/−^ mice.

Significant recent findings have revealed the important roles of endogenous inhibitors of gene expression in cardiovascular diseases, including atherosclerosis. Most previous reports have described the roles of these inhibitors in cancer, with some of these inhibitors being miRNAs, i.e., small (∼22 nt) RNA sequences that regulate and alter gene expression at the posttranscriptional level [41, 42]. The functions of macrophages, endothelial cells, and VSMCs are controlled by miRNAs, which thereby regulate the progression of atherosclerosis [43]. Smooth muscle cell differentiation is controlled by the downregulation of miRNA-145, and this process also promotes the formation of lesions [44]. Furthermore, the transfer of miRNA-126 from apoptotic endothelial cells to microbubbles signals a need for cellular repair [45]. Atherosclerotic lesions and inflammatory macrophages signify the presence of increased concentrations of miR-155 [45]. A previous study identified miR-145 as the most abundant miRNA in differentiated VSMCs. However, this miRNA is rapidly downregulated in subcultured dedifferentiated VSMCs, although its expression in these cells can be stimulated by exposure to platelet-derived growth factor [46]. In our study, we observed a decrease in the expression of PDCD4 miRNA and an increase in the expression of miRNA-21 in aortic tissues from mice treated with bosentan.

In conclusion, we investigated the expression of miRNA-21 and its target PDCD4 during disease progression in an ApoE^−/−^ mouse model of atherosclerosis induced by a high-fat diet. Notably, bosentan alleviated the adverse aortic effects of the high-fat diet, such as intimal thickening and deposition. Bosentan also decreased the expression of PDCD4, caspase 3, and Bax and increased the expression of Bcl-2 in aortic tissues, increased the expression of miRNA-21, and decreased the expression of PDCD4 mRNA. Our results thus indicated that the protective effect of bosentan against atherosclerosis in ApoE-deficient mice is mediated by miRNA-21.

## Figures and Tables

**Table 1:** The body weights of mice in the control, ApoE^−/−^, and bosentan groups at baseline and after 12 weeks of treatment.

Group	Number of animals	Body weight (g)	Body weight (g)
		At week 6	At week 12
Control	10	15.98 ± 0.19	28.20 ± 0.82
ApoE^–/–^	10	15.95 ± 0.29	33.62 ± 0.57^*∗*^
ApoE^–/–^ + bosentan	10	15.99 ± 0.20	32.04 ± 0.72^*∗*#^

^*∗*^ *P* < 0.05 vs. control group; ^#^*P* > 0.05 vs. ApoE^−/−^, group; *n* = 10/group.

**Table 2:** Effect of bosentan on the blood lipid concentrations in mice.

Group	*n*	Blood lipid concentrations (mmol/L)			
		TG	TC	HDL	LDL
Control	10	0.556 ± 0.033	1.740 ± 0.071	1.242 ± 0.057	0.164 ± 0.018
ApoE^–/–^	10	1.210 ± 0.129^*∗*^	19.36 ± 1.892^*∗*^	2.760 ± 0.246^*∗*^	5.310 ± 0.560^*∗*^
ApoE^–/–^ + bosentan	10	1.520 ± 0.338^*∗*#^	16.44 ± 1.032^*∗*#^	2.554 ± 0.292^*∗*#^	4.882 ± 0.329^*∗*#^

^*∗*^ *P* < 0.05, compared with the control group; ^#^*P* > 0.05, compared with the ApoE^−/−^ group; *n* = 10/group. TG, triglycerides; TC, total cholesterol; HDL, high-density lipoprotein; LDL, low-density lipoprotein.

## Data Availability

The data used to support the findings of this study are available from the corresponding author upon request.
